# Parity and All-cause Mortality in Women and Men: A Dose-Response Meta-Analysis of Cohort Studies

**DOI:** 10.1038/srep19351

**Published:** 2016-01-13

**Authors:** Yun Zeng, Ze-min Ni, Shu-yun Liu, Xue Gu, Qin Huang, Jun-an Liu, Qi Wang

**Affiliations:** 1 Department of Epidemiology and Biostatistics, School of Public Health, Tongji Medical College, Huazhong University of Science and Technology, Wuhan, Hubei Province, China; 2 Department of Social Medicine and Health Management, School of Public Health, Tongji Medical College, Huazhong University of Science and Technology, Wuhan, Hubei Province, China; 3 Women and Children Medical Center of Jiang-an District, Wuhan, Hubei Province, China; 4 Department of Medical Rehabilitation, Union Hospital, Tongji Medical College, Huazhong University of Science and Technology, Wuhan, Hubei Province, China

## Abstract

To quantitatively assess the association between parity and all-cause mortality, we conducted a meta-analysis of cohort studies. Relevant reports were identified from PubMed and Embase databases. Cohort studies with relative risks (RRs) and 95% confidence intervals (CIs) of all-cause mortality in three or more categories of parity were eligible. Eighteen articles with 2,813,418 participants were included. Results showed that participants with no live birth had higher risk of all-cause mortality (RR= 1.19, 95% CI = 1.03–1.38; *I*^*2*^ = 96.7%, *P* < 0.001) compared with participants with one or more live births. Nonlinear dose-response association was found between parity and all-cause mortality (*P* for non-linearity < 0.0001). Our findings suggest that moderate-level parity is inversely associated with all-cause mortality.

Multiple studies have shown that reproduction factors may affect the health of women in later life^1^,^2^,^3^,^4^,^5^,^6^. A recent meta-analysis showed that high parity is associated with reduced risk of pancreatic cancer^7^. A systematic review^8^ to studies of relationship between parity and mortality among women published before 2003 found that parity has long-term effect on women’s mortality. The report demonstrated that the relationship varied regarding to various populations and fertility patterns. We carefully scrutinized the studies listed in the review and included those that met our inclusion criteria. Studies on men also found that men’s mortality risk was associated with parity due to different socioeconomic status and parity-related habits and behaviors^9^.

“Antagonistic pleiotropy” and “disposable soma” theories, derived from experiments on both male and female *Drosophila melanogaster*, described the existence of a trade-off between reproductive and somatic maintenance, suggesting that parity is associated with increased risk of death^10^,^11^,^12^,^13^. However, other studies have presented contradictory findings, where parity is negatively associated with all-cause mortality^2^. Parity-related habits and behaviors, such as smoking less, drinking less, and exercising more^2^,^4^,^9^ may contribute to reduction in total mortality. Another study suggested that parity was associated with decreased risk of death from respiratory diseases and cancers^6^. Some researchers have proposed a U-shaped association between parity and mortality risk^1^,^6^. Recently, a meta-analysis^14^ on studies published between 2005 and 2009 was conducted using an iterative strategy to search articles and a meta-regression model to assess the non-linear association of parity and all-cause mortality. The results indicated that moderate number of parity was associated with decreased mortality risk in both women and men and low or high level of parity was associated with increased mortality risk in them. The means of hazard ratios stratified by the number of covariates were estimated without 95% confidence intervals. The non-linear association was tested with parsimonious regression model which failed to provide 95% confidence intervals of hazard ratios. Therefore, we conduct a dose-response meta-analysis on studies published before 24 April 2015, including studies published in recent years to assess the association between parity and all-cause mortality among women and men quantitatively. We carefully scrutinized the studies listed in the meta-analysis mentioned above, and included those that meet our inclusion criteria.

## Results

### Literature search

**Figure 1:**
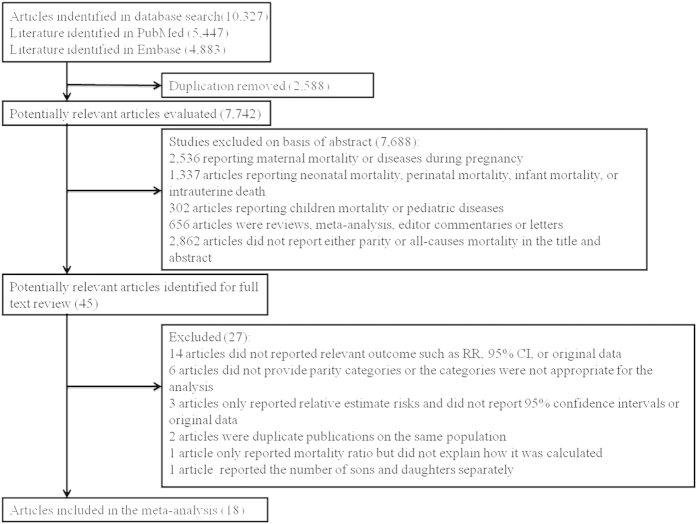
Selection of studies for inclusion in a meta-analysis of parity and all-cause mortality.

Results of the literature search and selection are shown in Fig. 1. We identified 5,447 and 4,883 articles from PubMed and Embase databases, respectively. Duplicates and papers that did not take parity as an exposure or those that did not related to parity and all-cause mortality were excluded. This left us with 45 articles for full-text review. A total of 27 articles were excluded after full-text review for the following reasons: (1) 14 did not report relevant outcome such as RR, 95% CI, or original data; (2) six did not provide parity categories or the categories were not appropriate for the analysis; (3) three only reported relative estimate risks and did not report 95% confidence intervals or original data; (4) two were duplicate publications on the same population; and (5) one only reported mortality ratio but did not explain how it was calculated; (6) one reported the number of sons and daughters separately. The remaining 18^1^,^2^,^3^,^4^,^5^,^6^,^9^,^15^,^16^,^17^,^18^,^19^,^20^,^21^,^22^,^23^,^24^,^25^ articles had a total of 2,813,418 participants and were included in the meta-analysis.

### Study characteristics

Table 1 shows the characteristics of the studies included in the meta-analysis. The sample size of participants ranged from 718 to 822,593. Eleven studies were conducted on women only, two studies were on men only, and five were on both women and men. The ages of female and male participants ranged from 23.8 to 100 years and 35 to 90 years, respectively. A total of five studies were conducted in the USA, two in Norway, three in Israel, and one each in UK, Netherland, Germany, Finland, Bangladesh, Japan, and Australia. Duration of follow-up in the included studies ranged from 2 to 42 years with a median of 18.7 years. Adjustment for confounding factors had not been done in two of the involved studies, but the other studies had controlled for factors such as age, marital status, education, smoking, drinking, socioeconomic status, age at first birth, and age at menopause. All studies had quality scores ranging from 6 to 9; four of them were of moderate quality and 14 were of high quality.

### All-cause mortality related to parity levels

**Figure 2:**
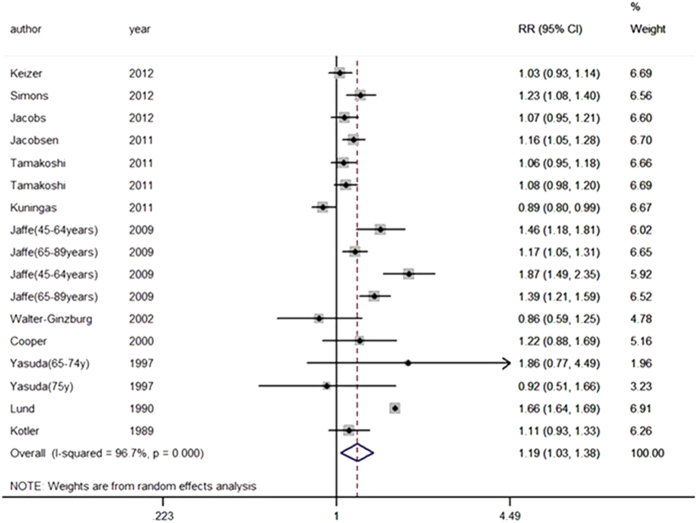
Pooled risk estimate for all-cause mortality among participants with no live birth compared with participants with one or more live births.

**Figure 3:**
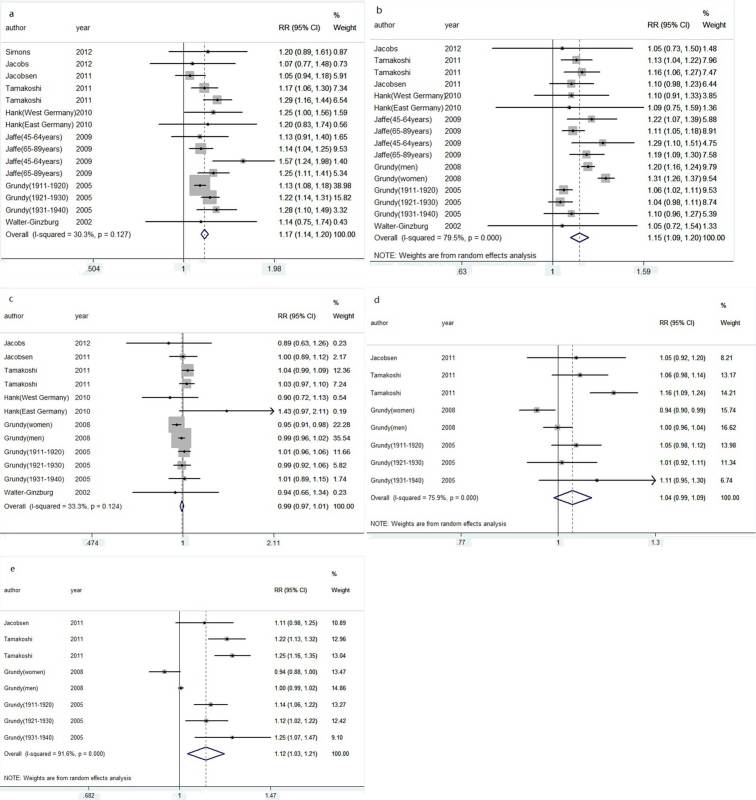
Pooled risk estimates for all-cause mortality among participants with zero (**a**), or one (**b**), or three (**c**), or four (**d**) or five or more (**e**) live births compared with participants with two live births.

Relationships between parity and all-cause mortality in women and men are shown in Table 2 and Figs 2, 3. The pooled RR for participants with no live birth was 1.19 (95% CI = 1.03–1.38; *I*^*2*^ = 96.7%, *P* < 0.001) compared with that with 1 or more live births (Fig. 2). Begg’s test and Egger’s test revealed evidence of publication bias in the studies. The trim and fill method was used to recalculate the pooled RR, and results indicated that the imputed RR was identical to the original RR. In addition, no missing studies imputed in the contour enhanced the funnel plot.

Compared with that of two live births, the pooled RRs of all-cause mortality were 1.17 (95% CI = 1.14–1.20; *I*^*2*^ = 30.3%, *P* = 0.127) for null parity, 1.15 (95% CI = 1.09–1.20; *I*^*2*^ = 79.5%, *P* < 0.001) for 1 live birth, 0.99 (95% CI = 0.97–1.01; *I*^*2*^ = 33.3%, *P* = 0.124) for 3 live births, 1.04 (95% CI = 0.99–1.09; *I*^*2*^ = 75.9%, *P* < 0.001) for 4 live births, and 1.12 (95% CI = 1.03–1.21; *I*^*2*^ = 95.7%, *P* < 0.001) for 5 or more live births (Fig. 3). No evidence of publication bias was detected using Egger’s test or Begg’s test. However, when five or more live births were compared with two live births, Begg’s test indicated no publication bias while Egger’s test indicated otherwise. We used the trim and fill method to recalculate the pooled RR. Results showed that two studies were imputed to enhance the funnel plot, but the imputed RR was identical to the original RR, validating the robustness of the result.

### Subgroup analyses

To explore the potential source of statistical heterogeneity among the studies and assess the stability of the results, we conducted subgroup analyses by country, sex, quality score, duration of follow-up period, number of participants, and number of cases. Results of subgroup analyses are shown in Table 3.

Subgroup analysis by country, sex of participants, and number of participants presented similar pooled RRs of all-cause mortality in relation to null parity compared with one or more live births. However, pooled analysis of the studies in Netherlands and Japan, with quality scores below 8 points, with duration of follow-up longer than 15 years, with 10,000 or less participants, or with 500 or fewer number of cases revealed that null parity was not associated with increased all-cause mortality compared with a parity level of one or more live births. Both women and men with one live birth had higher risk of all-cause mortality than those with two live births. However, the same pattern was not found among Germans and Americans, studies with quality scores below 8 points, and studies with 10,000 or fewer participants. Participants with five or more live births had increased all-cause death compared with those with two live births, except for Norwegians or studies with follow-up duration longer than 15 years.

Statistical heterogeneity in all-cause mortality comparisons between one and two live births and between five or more and two live births were mainly from studies performed in Norway and Israel. When Norwegian studies were excluded, similar findings with reduced statistical heterogeneity were observed. Statistical heterogeneity among all-cause mortality comparisons between four live births and two live births came mainly from studies in Norway and Japan. Studies conducted in Israel and Norway were the main sources of statistical heterogeneity among all-cause mortality comparisons between null parity and one or more live births. No evidence of significant statistical heterogeneity was observed after these studies were excluded. In addition, the pooled RR was not significantly altered.

### Dose-response association between parity and all-cause mortality

**Figure 4:**
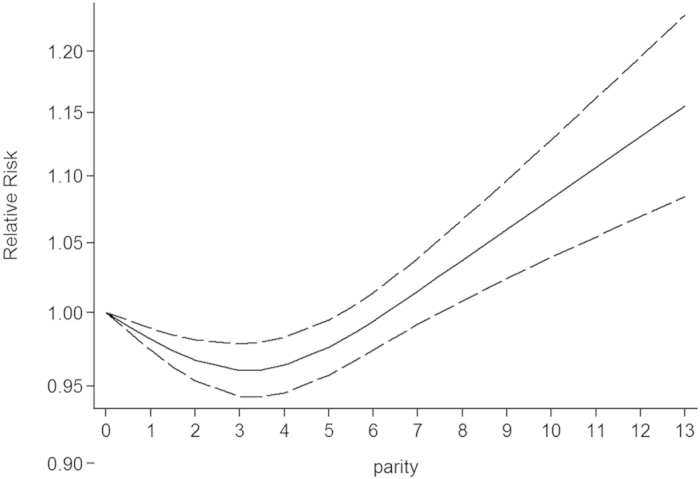
Results of dose-response analyses of parity and all-cause mortality. Parity was modeled with restricted cubic splines in a multivariate random-effects dose-response model. Null parity served as the reference group. The RRs are plotted on the log scale. Dashed lines represent 95% CIs for the spline model.

Eleven articles were included in our dose-response meta-analysis. Statistically significant evidence of non-linear association was found between parity and all-cause mortality (*P* = < 0.0001 for non-linearity; Fig. 4). Compared with null parity, the pooled RRs of all-cause mortality were 0.98 (95% CI = 0.97–0.99) for one live birth, 0.97 (95% CI = 0.95–0.98) for two live births, 0.96 (95% CI = 0.94–0.98) for three live births, 0.96 (95% CI = 0.95–0.98) for four live births, 0.98 (95% CI = 0.96–0.99) for five live births, 0.99 (95% CI = 0.97–1.01) for six live births. The lowest risk reduction for all-cause mortality (0.96) was observed for 3–4 live births. In sensitivity analysis, we excluded categories of more than five live births^2^. The nonlinear association between parity and all-cause mortality was not materially changed after excluding the categories with parity number greater than 5 (*P* < 0.0001 for non-linearity).

## Discussion

In this meta-analysis, the association between parity and all-cause mortality was investigated. Evidence of a nonlinear dose-response association between parity and all-cause mortality was found. Increased number of parity was associated with decreased risk of all-cause mortality, and the lowest risk reduction for all-cause mortality was observed among subjects with three to four live births. In addition, subgroup analysis revealed that statistical heterogeneity was affected mainly by the study locations.

The exact biological mechanisms underlying the nonlinear dose-response association between parity and risks of all-cause death in humans have not been fully understood. For men, the declining trend of nonlinear association of total death risk with increasing number of parity may be explained by parity-related habits and behaviors. It has been reported that participants with at least one live birth are more likely to have healthy behaviors, such as smoking less, drinking less, and exercising more compared with those with no live birth^2^,^4^,^9^. Such healthy behaviors may contribute to the reduced total mortality observed among the former compared to the latter. Another possible explanation is the decreased risk of death from respiratory diseases and cancers among both males and females^6^.

As for women, the physical changes related to reproduction may play an important role in reducing all-cause mortality. It is well-known that the serum estrogen levels of women can be elevated during pregnancy. In addition, both *in vitro* and *in vivo* studies have suggested that endogenous estrogens may protect women from pancreatic cancer, which is one of the leading causes of cancer-related deaths^26^,^27^. Experiments in rats have shown that estrogen inhibits the growth of preneoplastic pancreatic lesions and transplanted pancreatic carcinoma^28^,^29^. Additionally, parity is inversely associated with the risk of breast cancer among women^30^. Possible mechanisms for this include change in gene expression levels, variation in estrogen sensitivity, and change in the reaction of stem cells to estrogen^31^.

However, dose-response meta-analysis revealed that the total mortality risk was not further reduced by high parity (e.g., six or more live births). For men, people with high parity possibly have lower socioeconomic status, and risk of death is consequently higher because of poor access to healthcare services^1^. “Antagonistic pleiotropy” and “disposable soma” theories derived from experiments on both male and female *Drosophila melanogaster* describe a trade-off between reproductive and somatic maintenance and suggest that parity may be associated with increased risk of death^10^,^11^,^12^,^13^. For women, besides the socioeconomic factors mentioned above, other factors during pregnancy may increase the risk of death. High-parity-related all-cause death risk is proposed to be related to increased risk of cardiovascular diseases among women. Pregnancy may result in perturbations in carbohydrate metabolism in women, leading to decreased glucose tolerance, increased insulin secretion, and insulin resistance^1^. Such changes increase the mortality caused by cardiovascular diseases. A prospective cohort study of 12,055 women in Finland showed that increased total mortality in high-parity groups was mainly attributed to increased mortality associated with cardiovascular diseases^5^. In addition, physical and psychological stress arising from pregnancy and childbearing may also increase the risk of death, especially among people with high parity (e.g., six or more live births)^16^.

Our dose-response meta-analysis suggested that a J-shaped nonlinear association exists between parity and all-cause mortality in both women and men, which is consistent with previous studies^1^,^4^,^5^,^20^. Some results of subgroup analysis were inconsistent with those of non-stratified analysis possibly due to low quality and/or small sample size of the studies involved. For example, there were only two reports about all-cause mortality for Germans with one live birth compared to those with two. Subgroup analysis on such studies should be prevented because of limited data.

The observed statistical heterogeneity among studies could be attributed mainly to the diversity of study locations. Studies on Norwegians, Israelites, and Japanese showed large statistical heterogeneity. The larger proportions of higher-order planned births in more recent Norwegian cohorts because of higher availability of contraception, legal abortion, and “family friendly” policies than in earlier Norwegian studies may be a plausible reason for the large statistical heterogeneity observed among Norwegian studies^2^. In Israel, parity is related to religious belief, i.e., a large family size is a marker of religiousness, which may explain the large proportions of high parity in Israel compared with other countries. Thus, different proportions of parity arising from religious, social or cultural factors may be another reason for the statistical heterogeneity observed among studies. The declining fertility rate of Japan^22^ may also have contributed to the statistical heterogeneity observed here.

Our study has several strengths. First, most studies included in the analysis were cohort studies of large sample sizes and with long follow-up durations. This significantly minimized selection bias and considerably increased statistical power to detect potential association between parity and all-cause mortality. Second, subgroup and sensitivity analyses were used to investigate the source of statistical heterogeneity observed in our findings in great detail. Third, the previous meta-analysis^14^ shown a non-linear association between parity and all-cause mortality through a parsimonious regression model while did not report the 95% confidence intervals of hazard ratios; and in our study a dose-response meta-analysis was performed to quantitatively assess the association between parity and all-cause mortality and calculate hazard ratios with their 95% confidence intervals. Furthermore, in the previous meta-analysis^14^ the risk ratios were stratified by the number of covariates, while in this study the dose-response association was modeled after adjustment for many covariates, improving the precision and accuracy of our findings.

Our study has several limitations that should be addressed. First, non-marital birth or childbirth in previous marriage may be misclassified as null parity. This may have caused underestimation of the level of parity-related all-death risk. As for studies not reporting the precise number of births, we utilized categories of number of children for analysis in order to minimize misclassification. Additionally, biological parenthood and step-parenthood were not described in detail and were reported separately, which may also have reduced the risk estimate. However, Keizer *et al*. reported that a small proportion of step-parenthood was included in the population they studied, and that step-parenthood did not alter their findings on parity-related mortality among males^9^. Therefore, any effect of this limitation is likely to be minimal. Second, most of the studies involved in our analysis adjusted for many covariates, but some did not adjust for important confounding factors such as socioeconomic status, alcohol intake, smoking, education, chronic condition, and age at first birth. Third, most of the studies focused on the elderly, which may have led to survivor bias and underestimation of association between parity and all-cause mortality. Nevertheless, a 42-year follow-up cohort study^1^ suggested the presence of association between parity and risk of death among all participants, which validates the inferences drawn in the current study.

In conclusion, results of our meta-analysis suggest that an association exists between parity and all-cause mortality. Low to moderate parity is associated with decreased risk of total death in both women and men. People with 3–4 live births have the lowest risk of total death. More prospective studies that control for all major confounding factors as well as studies exploring the biological mechanisms underlying the effect of parity on death risk are still needed.

## Materials and Methods

### Literature search and selection

Studies published before 24 April 2015 were searched in the PubMed and Embase databases. The search was limited to studies carried out in humans, and the following key words and Medical Subject Headings were used: (“parity” or “number of live birth” or “number of children” or “parities” or “number of deliveries” or “number of living birth” or “number of live births” or “number of livebirth” or “number of kids” or “number of kid”) AND (“mortality” or “death” or “mortalities” or “dying” or “death rate” or “death rates” or “fatality rate” or “fatality rates” or “rate death” or “rates death” or “deaths”). To find additional references, we manually searched the bibliographies of all retrieved studies and selected all relevant publications. Only studies published in English were included. The following were excluded: conference literature, unpublished literature, and gray literature produced at all levels of government, academics, business, and industry in print and electronic formats, but not controlled by commercial publishers^32^.

Published studies were included in the meta-analysis if they met the following criteria: 1) the study was either prospective or had a historical cohort study design; 2) parity was the subject of interest; 3) the outcome was all-cause mortality; 4) the investigators reported relative risk (RR), hazard ratio (HR), or odds ratio (OR) and the corresponding 95% confidence intervals (CIs) for each parity category. If multiple publications had the same population as subjects, we included the most recent and most complete study. Two independent investigators (YZ and ZN) conducted initial screening of all titles or abstracts and then evaluated all potentially relevant articles based on full-text reviews.

### Data extraction

Two investigators (SL and XG) independently performed eligibility evaluation, data extraction, and quality assessment of each eligible study. All disagreements were discussed and resolved by consensus. The following data were extracted from each study: surname of the first author, publication year, study location, sex, and age range of the studied population, duration (in years) of follow-up, number of deaths, size of cohort, parity assessment, outcome assessment, parity category, RR or HR or OR and the 95% CI for each parity category, and factors adjusted in the report. If multiple estimates of the association were available, we extracted the estimate and adjusted for most covariates. If no adjusted risk estimate was presented, we used the crude risk estimate. If no risk estimate was reported, we calculated the crude risk estimate and its 95% CI using raw data provided with the article.

### Quality assessment

Quality assessment was conducted according to the Newcastle–Ottawa quality assessment scale^33^,^34^, which is a validated scale for cohort studies in meta-analysis. The highest score was 9, and scores of 0–3, 4–6, and 7–9 indicated low, moderate, and high quality studies, respectively.

### Statistical analysis

In our meta-analysis, we used pooled RRs and their 95% CIs to measure the association between parity and all-cause mortality. Any result stratified by age and sex was treated as a separate report.

Statistical heterogeneity among studies was evaluated using Cochran’s Q test and *I*^*2*^ statistic^35^. *I*^*2*^ values of 25%, 50%, and 75% were assigned to low, moderate, and large statistical heterogeneities, respectively^35^. The fixed effect model was adopted when *I*^*2*^was <50%, whereas the random effect model was used when *I*^*2*^was ≥50%^36^. To determine the source of statistical heterogeneity, we conducted subgroup analyses stratified by study location, sex, duration of follow-up (in years), size of cohort, and study quality.

For dose-response analysis, we used the two-stage random-effect dose-response meta-analysis method proposed by Greenland and Longnecker^37^,^38^,^39^,^40^ to determine the potential curve linear association between parity and all-cause mortality. This analysis was done by modeling parity using restricted cubic splines with three knots at 10%, 50%, and 90% percentiles of the distribution^39^,^41^. First, a restricted cubic spline model with two spline transformations (three knots minus one) was fitted in consideration of the correlation within each set of published RR^40^. Second, the restricted maximum likelihood method was used to combine the specific estimates of each study in the multivariate random effect meta-analysis^42^. An overall *P* value was calculated by testing whether the two regression coefficients were simultaneously equal to zero. A null hypothesis stating that coefficient of the second spline is equal to zero was created to test the non-linearity. Original parity levels reported in each study were used in the dose-response analysis. If parity intervals were provided, the midpoint between the lower and upper bounds of parity interval was regarded as the corresponding parity dose. In cases that involve an open-ended upper interval, we assumed that the category exhibited the same amplitude as the adjacent interval^7^,^43^.

Moreover, sensitivity analyses were conducted to examine the influence of specific studies or high parity categories on overall RRs by excluding specific studies or data points with parity level above five live births. Publication bias was evaluated by conducting Egger’s and Begg’s regression tests^44^,^45^. The STATA software (version 11.0; Stata Corporation, College Station, Texas, USA) was used to conduct all statistical analyses. All tests were two-sided with a significance level of 0.05.

## Additional Information

**How to cite this article**: Zeng, Y. *et al*. Parity and All-cause Mortality in Women and Men: A Dose-Response Meta-Analysis of Cohort Studies. *Sci. Rep*. **6**, 19351; doi: 10.1038/srep19351 (2016).

## Figures and Tables

**Table 1:** Characteristics of cohort studies of Parity and All-cause mortality included in the Meta-Analysis.

Authors, year	Country	Sex, age	Follow-up period	Follw-up length	Case/subject	Parity	Adjustment variables	Exposure assessment	Outcome assessment	Quality score
Dior *et al*., 2013 (1)	Israel	Women 23.8–60.9y	1964–2005	42	2,766/40,454	1,2–4,5–9,10+	Age at first birth, mother’s origin, socioeconomic status, diabetes mellitus, gestational diabetes mellitus, toxaemia, hypertension, smoking, multiple pregnancies, Cesarean sections	Israeli Population Registry	Israeli Population Registry	9
Jacobs *et al*., 2012 (21)	USA	Women 50–96y	1984–2007	24	707/1,294	0,1,2,3,4+	Age, years postmenopause, BMI, and HDL cholesterol	Interviewer administered questionnaire	During follow-up	9
Simons *et al*., 2012 (6)	Australia	Women 69.6y	1988–2004	17	683/1,571	0.1,2,3,4,5,6+	Alcohol intake, smoking, peak expiratory flow, physical disability, self-rated health and atrial fibrillation, hypertension, diabetes, BMI	Self-administered questionnaire	Death record	8
Keizer.al., 2012 (9)	Netherlands	Men 45–75y	1991–2007	17	1,551/4,961	0,1,2–3,4+	Age, chronic conditions, occupational class, education, drinking, smoking, live with partner	Interviewer administered questionnaire	Statistics Netherlands	9
Jacobsen *et al*., 2011 (18)	USA	Women 27–100y	1976–1988	13	3,122/12,688	0,1,2,3,4,5+	Marital status	Self-administered questionnaire	ICD-9 codes	6
Kuningas *et al*., 2011 (23)	Netherlands	Women 68y	1990–2008	19	1,116/3,575	0,1,2–3,4+	education and age at baseline	Self-administered questionnaire	During follow-up	6
Tamakoshi *et al*., 2011 (22)	Japan	Women Men 40–79y	1988–2006	14.4	18,807/110,792	0,1,2,3,4,5+	Age, residential area group, marital status, smoking status, alcohol consumption status, walking hours, sleeping hours, consuming green-leafy vegetables, BMI, education, mental stress, disease history and employment status	Self-administered questionnaire	Death certificates from the Director-General of the Prime Minister’s Office	8
Hank *et al*., 2010 (3)	Germany	Women 50–99y	1984–2007	24	Not available/9,514	0,1,2,3,4+	Age, marital status, education, homeowner, household income	Interviewer administered questionnaire	During follow-up	9
Jaffe *et al*., 2009 (4)	Israel	Men 45–89 y women 45–89 y	1995–2004	10	Men 13,309/71,733 women 6,128/62,822	1,2,3–4,5–7,8+	Age, origin, education, rooms	Census, interviewer administered questionnaire	Death record from the Israel Central Bureau of Statistics	9
Grundy *et al*., 2008 (2)	Norway	Men 45–68y women 45–68y	1980–2003	24	Men 40,071/785,317 women 23,241/744,784	0,1,2,3,4,5+	Education, marital status	Census, interviewer administered questionnaire	Death record from the Central Population Register	9
Koski-Rahikkala *et al*., 2006 (5)	Finland	Women 49–83y	1966–2001	36	1,075/13,002	1,2–4,5–9,10+	Age, BMI, smoking, socioeconomic position, age at menarche, age at first birth	Self-administered questionnaire	Death record from the Population Registration Centre	8
Grundy *et al*., 2005 (16)	UK	Women 50–89y	1971–2000	30	29,329/87,477	0,1,2,3,4,5+	Age, marital status, social class, education, car access housing tenure, widowhood	Census, interviewer administered questionnaire	The Office for National Statistics Longitudinal Study	8
Hurt *et al*., 2004 (17)	Bangladesh	Men 45–90y women 45–71y	1982–1998	17	Men 4,394/14,803 women 1,939/20,383	0–2,3–5,6–8,9–11,12+	Age, time period, religion, education, occupation, area of residence, marital status	Interviewer administered questionnaire	Death record from the Health and Demographic Surveillance System	9
Walter-Ginzburg *et al*., 2002 (24)	Israel	Women Men 75y	1989–1997	9	813/2,400	0,1,2,3,4+	Unadjusted	Interviewer administered questionnaire	Death record from the National Death Registry	7
Cooper *et al*., 2000 (15)	USA	Women 63–81y	1990–1991	2	108/718	0,1–2,3–4,5+	Age, smoking, use of estrogen replacement therapy, age at menopause, surgical	Self-administered questionnaire	During follow-up	6
Yasuda *et al*., 1997 (25)	USA	Women 65y	1984–1988	5	149/806	0,1–2,3+	Perceived health status, activities of daily living impairment, number of chronic conditions, and years of education	Interviewer administered questionnaire	During follow-up	7
Lund *et al*., 1990 (20)	Norway	Women 25–84y	1970–1985	16	112,023/822,593	0,1+	Unadjusted	Self-administered questionnaire	Death record from the Central Population Register of the Central Bureau of Statistics	6
Kotler *et al*., 1989 (19)	USA	Men 35–64y	1965–1982	18	342/1,731	0,1–3,4+	Age, marital status, parenthood	Self-administered questionnaire	Death record from the California Death Registry	7

Abbreviations: BMI, body mass index.

**Table 2:** Meta-analysis of parity and all-cause mortality.

	No. of study	Model selected	Pooled RR	95% CI	P* value		Egger	Begg
						*I*^2^ (%)	*P*† value	*P*‡ value
0 vs. 1+	17	Random	1.19	1.03–1.38	<0.001	96.7	0.001	0.012
0 vs. 2	15	Fixed	1.17	1.14–1.20	0.127	30.3	0.215	0.843
1 vs. 2	16	Random	1.15	1.09–1.20	<0.001	79.5	0.521	0.620
3 vs. 2	12	Fixed	0.99	0.97–1.01	0.124	33.3	0.510	1.000
4 vs. 2	8	Random	1.04	0.99–1.09	<0.001	75.9	0.260	0.386
5+ vs. 2	8	Random	1.12	1.03–1.21	<0.001	91.6	0.036	0.536

Abbreviations: CI, confidence interval; RR, relative risk.

^*^P value for heterogeneity.

^†^*P* value for Egger’s test.

^‡^*P* value for Begg’s test.

**Table 3:** Stratified analysis on association of parity and all cause mortality.

Study Characteristic	No. of study	Model selected	RR	95% CI	P* value	*I*^2^ (%)
0 vs. 1+	17	Random	1.19	1.03–1.38	<0.001	96.7
Country						
Israel	5	Random	1.34	1.11–1.61	<0.001	80.0
USA	6	Fixed	1.13	1.05–1.21	0.703	0.0
Netherlands	2	Fixed	0.96	0.89–1.03	0.051	73.8
Japan	2	Fixed	1.07	0.99–1.15	0.805	0.0
Sex						
Women	11	Random	1.25	1.04–1.50	<0.001	96.8
Men	5	Random	1.13	1.03–1.24	0.041	59.9
Quality score						
>=8	9	Random	1.21	1.10–1.33	<0.001	80.2
<8	8	Random	1.14	0.88–1.49	<0.001	96.7
Duration of follow-up						
>15	6	Random	1.14	0.87–1.50	<0.001	98.3
<=15	11	Random	1.22	1.11–1.36	<0.001	72.5
No. of participants						
>10,000	8	Random	1.32	1.11–1.58	<0.001	96.7
<=10,000	9	Random	1.06	0.96 –1.16	0.014	58.3
No. of cases						
>500	13	Random	1.20	1.02–1.41	<0.001	97.4
<=500	4	Fixed	1.14	0.98–1.32	0.585	0.0
1 vs. 2	16	Random	1.15	1.09–1.20	<0.001	79.5
Country						
USA	2	Fixed	1.10	0.98–1.22	0.808	0.0
Israel	5	Fixed	1.15	1.11–1.21	0.283	20.7
Norway	2	Random	1.25	1.14–1.37	0.001	90.9
UK	3	Fixed	1.06	1.02–1.09	0.745	0.0
Germany	2	Fixed	1.10	0.93–1.30	0.966	0.0
Japan	2	Fixed	1.14	1.08–1.21	0.670	0.0
Sex						
Women	11	Random	1.14	1.06–1.23	<0.001	84.9
Men	4	Fixed	1.17	1.14–1.20	0.113	49.8
Quality score						
>=8	14	Random	1.15	1.10–1.21	<0.001	82.0
<8	2	Fixed	1.10	0.98–1.22	0.818	0.0
Duration of follow-up						
>15	8	Random	1.13	1.04–1.23	<0.001	89.5
<=15	8	Fixed	1.15	1.11–1.19	0.561	0.0
No. of participants						
>10,000	12	Random	1.15	1.10–1.21	<0.001	84.8
<=10,000	4	Fixed	1.08	0.84–1.25	0.994	0.0
4 vs. 2	8	Random	1.04	0.99–1.09	<0.001	75.9
Country						
UK	3	Fixed	1.04	0.99–1.10	0.579	0.0
Norway	2	Random	0.97	0.92–1.03	0.085	66.3
Japan	2	Random	1.11	1.02–1.21	0.074	68.7
Sex						
Women	6	Random	1.02	0.97–1.08	0.039	57.3
Men	2	Random	1.07	0.93–1.24	<0.001	93.5
Duration of follow-up						
>15	5	Random	1.00	0.96–1.05	0.067	54.5
<=15	3	Fixed	1.11	1.06–1.16	0.141	48.9
5+ vs. 2	8	Random	1.12	1.03–1.21	<0.001	91.6
Country						
UK	3	Fixed	1.14	1.09–1.21	0.496	0.0
Norway	2	Random	0.98	0.92–1.04	0.525	75.2
Japan	2	Fixed	1.24	1.17–1.30	0.661	0.0
Sex						
Women	6	Random	1.12	1.02–1.23	<0.001	84.7
Men	2	Fixed	1.01	1.00–1.02	<0.001	96.8
Duration of follow-up						
>15	5	Random	1.10	0.99–1.15	<0.001	86.1
<=15	3	Fixed	1.21	1.16–1.28	0.264	25.0

Abbreviations: CI, confidence interval; RR, relative risk.

^*^P value for heterogeneity.
